# The Battle Against Systemic Sclerosis: A Case Report of a Patient With Scleroderma Renal Crisis

**DOI:** 10.7759/cureus.117638

**Published:** 2026-10-08

**Authors:** Noor Elkurwi, Zeineb Garmeh, Qabas Elkurwi, Zahra H Abu Jawhar, Malgorzata Wajdlich

**Affiliations:** 1 Department of Nephrology, Hypertension, Transplantation, and Internal Medicine, Medical University of Lodz, Lodz, POL; 2 Department of Dermatology, Dammam Health Network, Dammam, SAU

**Keywords:** angiotensin-converting-enzyme inhibitors, glucocorticosteroids, raynaud's phenomenon, sclerodactyly, scleroderma renal crisis, systemic scleroderma, therapeutic plasma exchange

## Abstract

Systemic scleroderma (SSc) is an uncommon autoimmune disorder that causes excessive fibrosis of the skin and internal organs, especially the lungs, kidneys, and heart. Scleroderma renal crisis (SRC) is a rare and potentially fatal complication of SSc. In recent years, there has been an increasing interest in SSc treatment, but so far, the targeted management approach has been lacking.

We report a 44-year-old male patient with overlap syndrome (systemic scleroderma, Sjogren's syndrome, and myositis), who underwent treatment with therapeutic plasma exchange (TPE) and received hemodialysis, angiotensin-converting enzyme inhibitors, glucocorticosteroids, and cyclophosphamide. The patient presented prominent cutaneous scleroderma with sclerodactyly and Raynaud's phenomenon. Despite the initial stabilization of the patient's condition on the multimodal pharmacotherapy combined with TPE, he later died from multi-organ failure.

Although TPE performed during the early course of the disease has been reported as a potential adjunctive therapy, its efficacy and long-term benefits remain uncertain. Given the complexity of our patient's presentation, including scleroderma overlap syndrome and advanced, irreversible organ damage, it is difficult to determine whether TPE provided a meaningful clinical benefit.

## Introduction

Scleroderma, systemic sclerosis (SSc), is an autoimmune dysfunction of connective tissues [1]. It is more prevalent in women, with a female-to-male ratio of 5:1 and a peak age of onset of 30-50 years [2]. SSc involves vasculopathy and inflammatory changes along with progressive deposition of collagen and fibrotic changes; this results in thickening and stiffening of body organs, potentially progressing to renal crisis [3]. The location determines the type of SSc; CREST syndrome is associated with limited SSc, which stands for calcinosis, Raynaud's phenomenon, esophageal dysmotility, sclerodactyly, and telangiectasia [1]. Systemic sclerosis sine scleroderma (ssSSc) is a rare subtype characterized by the total or partial absence of typical cutaneous manifestations, where changes in the internal organs appear before changes in the skin [4].

SSc is associated with malfunctioning innate and adaptive immune systems. Excess autoantibodies are produced, which induce vasculopathy and scarring of the skin and internal organs [2]. It is also associated with a genetic predisposition such that non-human leukocyte antigen (HLA)-linked genes and alleles of major histocompatibility loci were found in SSc patients [1]. The progression of the disease results in decreased kidney perfusion and therefore develops into a scleroderma renal crisis (SRC). In addition, activated endothelial cells play a critical role in the development of SRC. A characteristic fibrotic 'onion-skin' can be found in the interlobular/arcuate renal arteries. This results in intimal hyperplasia, inducing critical narrowing and occlusion of the arterial lumen. The main indicators of the development of SRC are hypertensive crisis, new-onset anemia, thrombocytopenia, rapidly progressing skin thickening, and systemic inflammatory reaction [2]. SRC risk factors include anti-RNA polymerase III autoantibodies, tendon friction rub and synovitis, and steroid therapy [2].

The signs and symptoms of SSc are related to the affected organ. SSc initially starts at the fingers, hands, feet, and the face. It is less frequent in the forearms, upper extremities, chest, abdomen, and legs. Cutaneous SSc of the hands (i.e., sclerodactyly) causes fingers to take on a claw-like shape, distinctly limiting their motion. Sclerodactyly is often accompanied by the formation of ulcers and Raynaud's phenomenon, an overactive sympathetic response to cold and stress in the vessels of the digits, causing discoloration and sensory symptoms in the fingers, toes, the tip of the nose, and ears [2].

Secondary to skin involvement, the lungs are affected with interstitial lung disease (ILD) [3,5]. Assessment of cardiac involvement is difficult due to its complex presenting symptoms, but the most common observed presentation is heart failure with preserved ejection fraction. Renal involvement is not as common in SSc, yet it is a sign of disease progression, shown as SRC. An earlier diagnostic factor of SSc would be gastrointestinal involvement in the form of gastroesophageal reflux disease (GERD) and diarrhea [5]. A well-recognized association with scleroderma is overlap syndrome (OS). OS is a combination of autoimmune disorders characterized by at least two connective tissue disorders, with the most common presentation being SSc and Sjogren syndrome, polymyositis or dermatomyositis, rheumatoid arthritis, and systemic lupus erythematosus [6].

The diagnosis of SSc is based on autoantibody screening tests, such as immunofluorescence, enzyme-linked immunosorbent assay (ELISA), and immunoblot [7]. Additional tests are also performed to assess particular organ involvement and determine the severity of scleroderma, including electrocardiography, echocardiography, chest radiograph, nailfold capillaroscopy, computed tomography, pulmonary function test, complete blood count lab test, urine strip test, N-terminal prohormone of brain natriuretic peptide (NT-pro-BNP), and a biopsy of affected organs [7]. SRC can be the initial presenting characteristic of SSc and is diagnosed by laboratory abnormalities, sudden and severe increase in blood pressure (BP), progressive kidney excretory impairment, congestive heart failure, and/or hypertensive encephalopathy [8]. In a German case report from 1993, the diagnosis of SRC was made based on biopsy and antinuclear antibody test for SSc. The patient's skin was unremarkable at the time of admission, and SRC was the only presenting feature [9]. In the absence of skin thickening and/or scleroderma antibodies, SRC should not be excluded if there is an abrupt hypertensive crisis with kidney failure. The diagnosis of SSc can be delayed due to an atypical clinical presentation and/or the absence of classical antibodies [2].

Optimal SSc management is classified into pharmacological and non-pharmacological approaches. The most common drugs used in the early stage of the disease are immunosuppressants, such as cyclophosphamide, glucocorticosteroids, methotrexate, mycophenolate mofetil, hydroxychloroquine, and others [10]. The choice of immunosuppressive agent depends on the patient's tolerance to the drug and its side effects [3]. Another group of medications commonly used in SSc is angiotensin-converting enzyme (ACE) inhibitors [11]. These medications are used more commonly in SRC. Studies have shown that the administration of ACE inhibitors in SRC patients decreased one-year mortality by approximately 61% [8]. It is recommended to start with short-acting ACE inhibitors, such as captopril, and rapidly titrate the dose to reduce systolic BP by 20 mmHg within the first 24 hours, reaching an optimal BP of 120/70 mmHg in no more than 72 hours. Once the target BP is achieved, the short-acting ACE inhibitor can be substituted with a long-acting one. Although ACE inhibitors are the cornerstone of treatment once SRC develops, prophylactic use does not prevent SRC. The prevalence of SRC in patients using ACE inhibitors early in their diagnosis of SSc is 5-10% [8,12]. ACE inhibitors do not prevent SRC; however, they delay the progression of SRC and may even mask the symptoms of hypertensive response. It has also been found that prolonged therapy with high-dose corticosteroids (>15mg/day for >6 months) may increase the risk of SRC [13].

Non-pharmacological treatment includes hemodialysis and therapeutic plasma exchange (TPE). Hemodialysis is indicated when the patient's kidney function remains severely impaired. Although TPE lacks solid evidence to be effective in the treatment of scleroderma, several case reports suggested that TPE is beneficial. A 2015 case report from a patient with progressive SSc showed an improvement in condition, based on the regression of the Baseline Modified Rodnan Skin Score (MRSS), after 15 sessions of TPE with 2 liters of frozen plasma exchanged per session [14]. Another case report by Nagamura et al. demonstrated the positive effect of TPE on scleroderma with progressive ILD and SRC. The volume of exchange was 73.4ml/kg of fresh frozen plasma per session for a total of nine TPE sessions. The improvement was based on stabilization of lung fibrosis on imaging and improvement in lung function after two years of TPE sessions [13]. Harris et al. reviewed the effect of TPE from multiple case reports, and most of them concluded that there was a significant improvement in laboratory markers and symptoms of scleroderma after TPE regardless of the duration of treatment. However, TPE cannot be conclusively established as the treatment of choice for SSc because in all these studies, TPE was used along with other pharmacological treatments [15]. Some studies have shown, in fact, the benefits of combined therapy with an immunosuppressive agent, hemodialysis, and TPE. According to Suga et al., patients who underwent a combined therapy with prednisolone, cyclophosphamide, and double-filtration TPE showed a great reduction in scleroderma antibodies after almost one month of follow-up [16]. Nagamura's study also reported a switch to a combined ACE inhibitor and TPE therapy after initial resistance to cyclophosphamide [13]. Nonetheless, there is no universally recommended pharmacological treatment for scleroderma, and more studies are needed.

This case was previously presented as an abstract oral presentation at the Juvenes Pro Medicina Conference on May 14, 2022.

## Case presentation

A 44-year-old male patient with scleroderma diagnosed two years ago was admitted to the cardiology ward complaining of dyspnea lasting one week. Physical examination revealed peripheral edema, pulmonary congestion, and the presence of fluid in the right pleural cavity. The initial diagnosis was de novo heart failure with a reduced ejection fraction (EF) of 15% and deterioration of renal function. On echocardiography, cardiac amyloidosis was suspected. The laboratory results obtained during the initial evaluation by the cardiology department showed a serum creatinine level of 182.6 µmol/l and an estimated glomerular filtration rate (eGFR) of 37.1 ml/min. Parenteral furosemide was administered for the treatment of heart failure, and EF improved to 35%. However, the patient's kidney function progressively worsened, with serum creatinine increasing markedly and eGFR decreasing over the subsequent days. The patient's rapid clinical deterioration prompted referral to our nephrology department, where the diagnosis of normotensive SRC was made. Although his blood pressure was variable during hospitalization, the hypertensive response may have been pharmacologically attenuated by ongoing ACE inhibitor therapy. 

Past medical history included overlap syndrome (systemic scleroderma, Sjogren's syndrome, and myositis), Raynaud's phenomenon, anemia, bile reflux, secondary thrombocytopenia, tobacco abuse, liver insufficiency, left kidney cyst, and mycophenolate mofetil intolerance. The patient was on prednisolone 20 mg/day and methotrexate 20mg once a week for the treatment of scleroderma. Diagnosis of scleroderma was already made in 2021 when the symptoms of Raynaud's phenomenon in the fingertips first appeared during working hours in a dry-ice production factory. 

Upon admission, the patient was cachectic with a low body mass index of 16.6 kg/m2. His BP was 140/90 mmHg, heart rate was 80 bpm, and EF was 21%. Bilateral dull sounds were heard during lung percussion. The laboratory findings obtained upon admission to the nephrology department are summarized in Table 1. Physical inspection showed advanced scleroderma with thickening of the skin, most prominent in the hands and wrists, as well as limited facial expressions (Figure 1). The symptoms initially manifested as stiffness of the phalanges in both hands. On examination, the patient had difficulty grasping and manipulating objects. Scabs were visible on the skin of the dorsal side of the fingers at the metacarpophalangeal joints and proximal interphalangeal joints.

**Table 1:** Laboratory findings upon admission to the nephrology department WBC - white blood cells; RBC - red blood cells; eGFR - estimated glomerular filtration rate; CRP - C-reactive protein; AST - aspartate aminotransferase; ALT - alanine aminotransferase; LDH - lactate dehydrogenase; BNP - B-type natriuretic peptide; aPPT - activated partial thromboplastin time; INR - international normalized ratio

	Baseline	Reference range
Complete Blood Count		
WBC	7.65*10^3 /ul	4.00 - 11.00 *10^3 /ul
RBC	3.12*10^6 /ul	4.20 - 6.10 *10^6 /ul
Platelet	192,000 /ul	150,000 - 400,000 /ul
Hemoglobin	9.5 g/dl	14 - 18 g/dl
Hematocrit	30.9%	40 - 55%
Chemistry Panel		
Potassium	4.67 mmol/L	3.5 - 5.1 mmol/L
Sodium	136.7 mmol/L	136 - 146 mmol/L
Chloride	100.5 mmol/L	101 - 109 mmol/L
Serum creatinine	425.6 umol/L	64 - 104 umol/L
eGFR	14.1 ml/min/1.73 m	> 60 ml/min/1.73 m
Urea	13.6 mmol/L	2.8 - 7.2 mmol/L
CRP	28.8 mg/L	<5 mg/L
Procalcitonin	0.58 ug/L	<0.5 ug/L
AST	17.9 U/L	<50 U/L
ALT	11.1 U/L	<50 U/L
Bilirubin	6.3 umol/L	5 - 21 umol/L
Serum albumin	31.5 g/L	35 - 52 g/L
LDH	294.9 U/L	0 - 248 U/L
BNP	35,000 pg/ml	<100 pg/ml
Coagulation tests		
Prothrombin time	14.4 s	11 - 13.5 seconds
Prothrombin Index	77.1%	65 - 135%
aPTT ratio	1	0.8 - 1.2
INR	1.3	0.8 - 1.1

**Figure 1:**
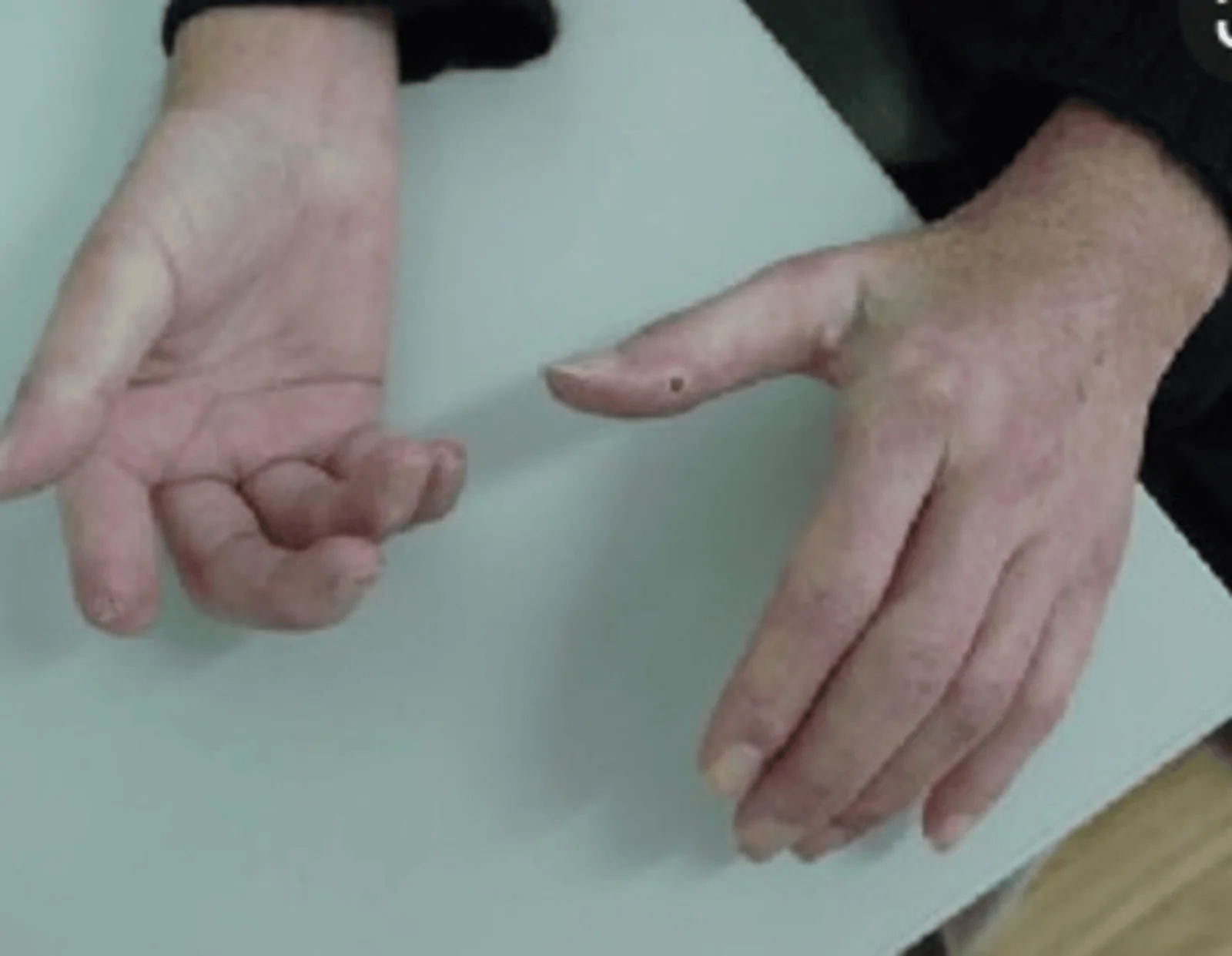
Presentation of skin thickening and finger curling noticeable on the patient's hand

A chest X-ray showed clear lungs with no focal lesions, no sign of pneumothorax or other pathologies. A central venous catheter (CVC) was visible in the left jugular vein. An enlarged heart silhouette with a normal aorta was observed (Figure 2). 

**Figure 2:**
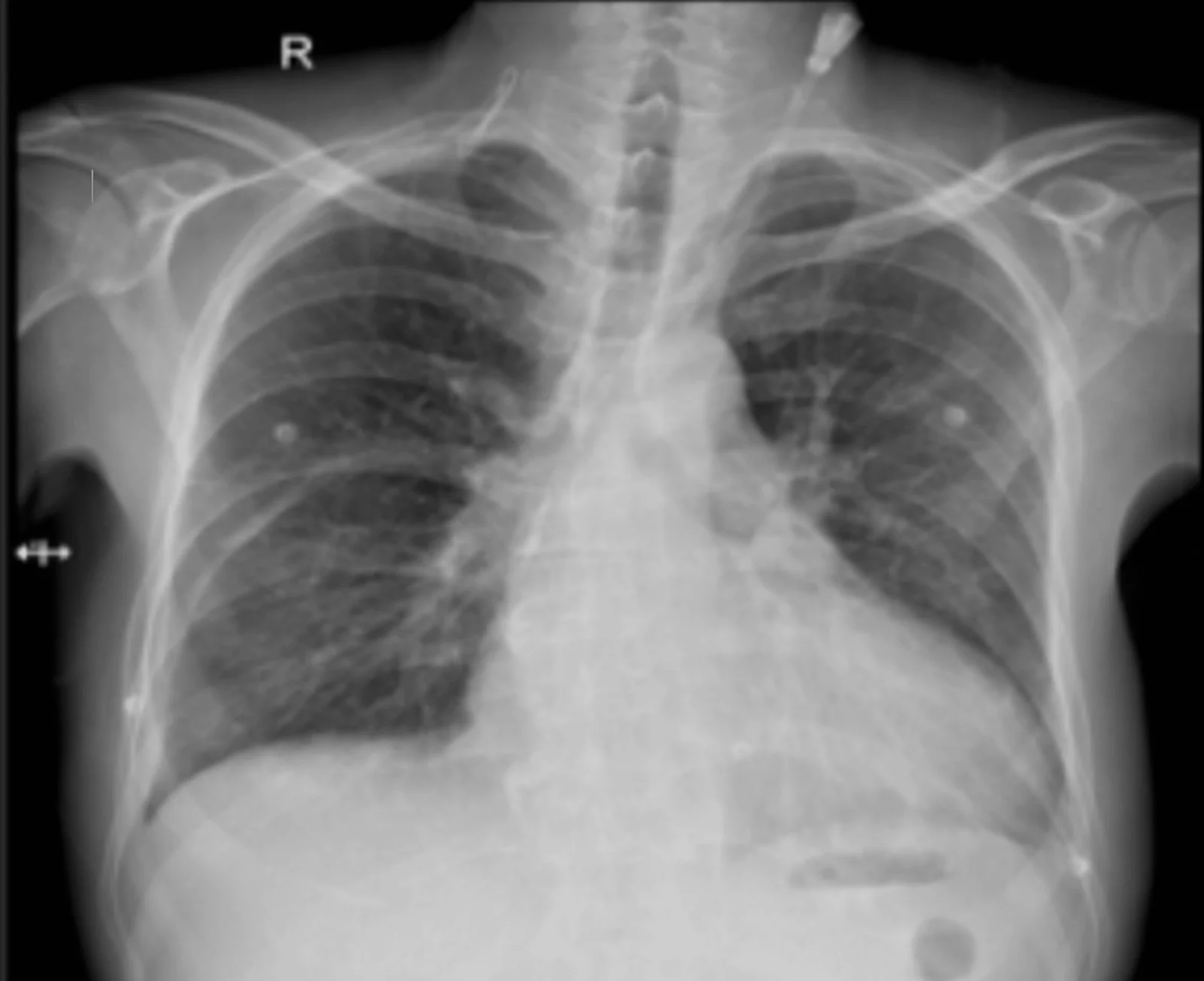
Chest X-ray ensured correct positioning of central venous catheter in superior vena cava. Pneumothorax and other complications excluded.

Abdominal ultrasonography confirmed a normal structure of the liver and spleen, without any focal changes. The intrahepatic bile duct and the gallbladder also appeared normal. The pancreas and kidneys were of normal size and appearance, with the exception of the well-demarcated cortical cyst 15 mm in diameter in the left kidney.

Angio-CT showed fluid in the pleural cavity, approximately 25 mm thick, on the right and 12 mm on the left side. Fine adhesions were visible in the apex of both lungs. Several emphysematous lesions, up to 6 mm, were observed in segments 2 and 3 of the right lung. Fibrotic changes were identified in segments 4 and 5 of the right lung (Figure 3). The heart was significantly enlarged. CVC was visible in the left internal jugular vein and superior vena cava. Pulmonary embolism was excluded. 

**Figure 3:**
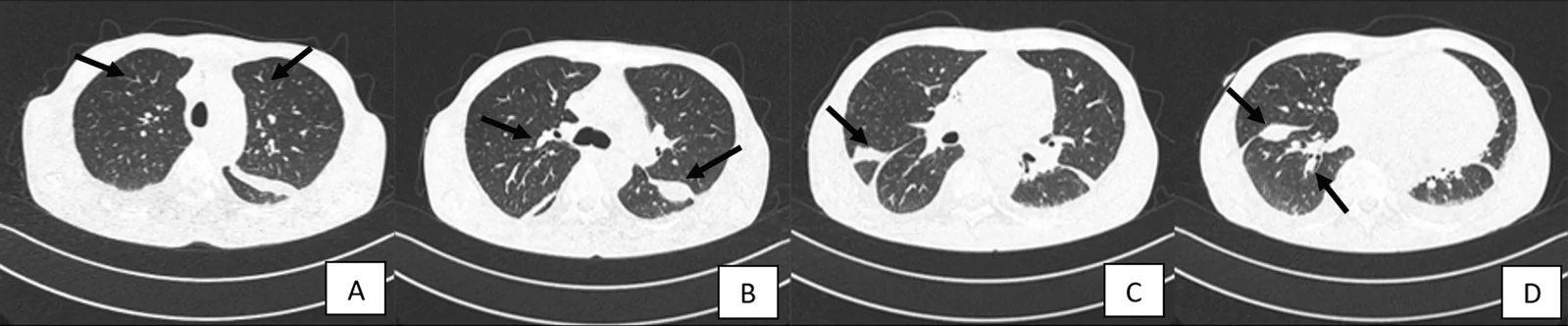
Axial CT chest images demonstrating bilateral pulmonary fibrosis (black arrows) (A) Upper thoracic (apical lung) level showing bilateral fibrotic changes. (B) Carinal level demonstrating bilateral fibrotic changes. (C) Main pulmonary artery/hilar level showing bilateral fibrotic changes. (D) Cardiac base level demonstrating extensive bilateral fibrosis, most prominent in the right middle lobe.

Throughout hospitalization, the patient presented fluctuating levels of C-reactive protein (CRP) (Figure 4). After insertion of the CVC, signs of inflammation and reddened skin around the jugular vein were noticeable. Treatment was started, and a slight reduction in the CRP value was observed. At the end of the second week of hospitalization, the patient experienced persistent greenish watery diarrhea, with a significant increase in CRP. He tested negative for the following: human immunodeficiency viruses (HIV), hepatitis C virus (HCV), cytomegalovirus (CMV), hepatitis B virus (HBV), *Salmonella*, *Shigella*, rotavirus, and norovirus. Additional microbiological tests indicated fungal infection of the gastrointestinal tract. During the last week of hospitalization, the patient developed dyspnea with an oxygen saturation of 93%. Crackles were heard in the left lung. Sputum culture confirmed *Candida albicans (C. albicans)*, *Acinetobacter baumannii (A. baumannii)*, and *Escherichia coli (E. coli)*, and a diagnosis of pneumonia was made. 

**Figure 4:**
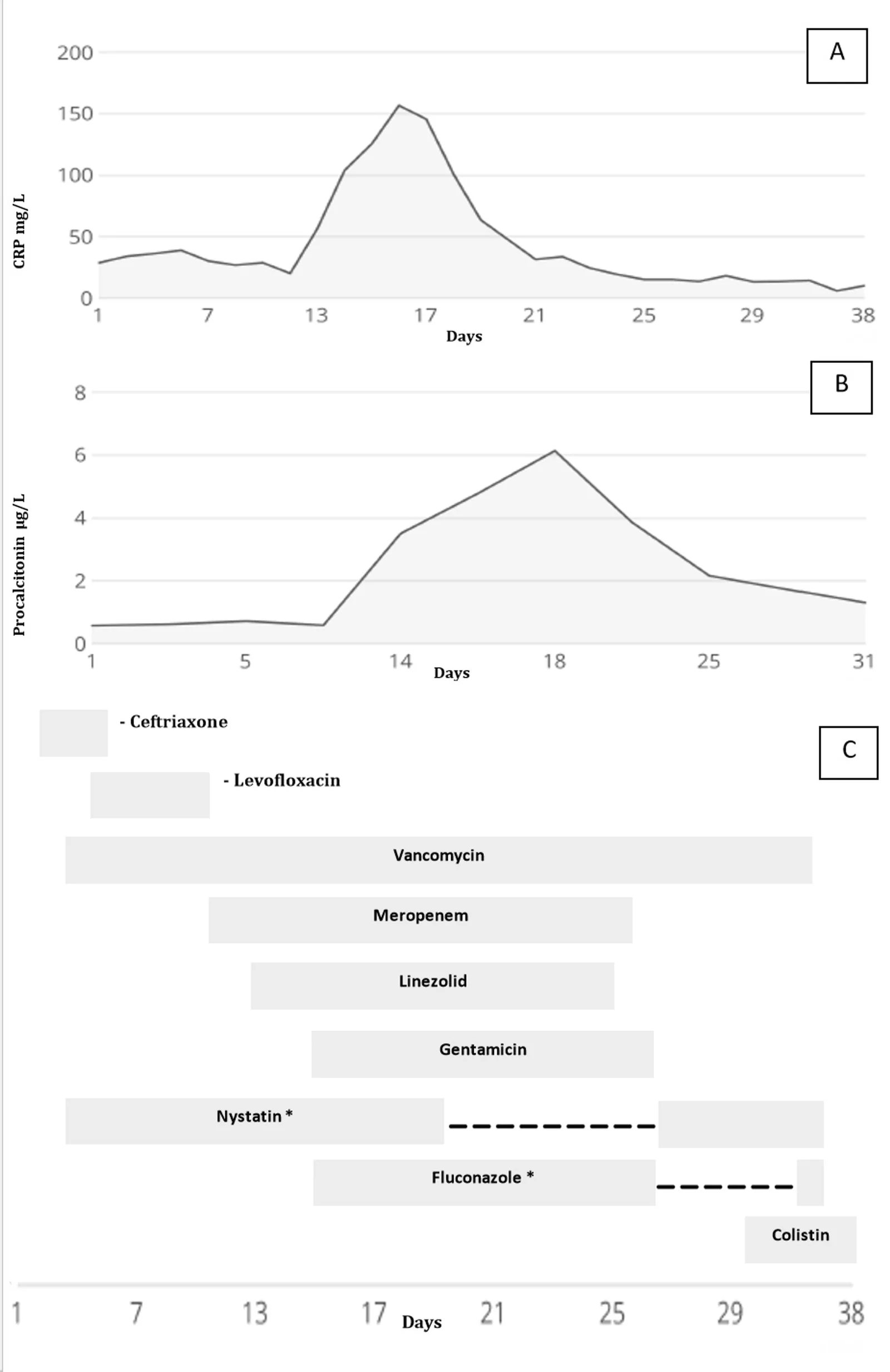
A) CRP levels throughout the hospitalization; B) Procalcitonin levels throughout the hospitalization; C) Antibiotics and antifungal intervention throughout the hospitalization * Administration of the drug was temporarily suspended. CRP - C-reactive protein

Pharmacological management

Upon admission to the nephrology department, methotrexate was discontinued due to its increased myelotoxicity in case of severely impaired kidney function and was replaced by daily oral cyclophosphamide 50 mg. Heart failure intervention with an ACE inhibitor and nebivolol was continued. Initially, he received short-acting captopril 12.5 mg three times a day, which was replaced by a long-acting ramipril 10 mg once a day. After consultation with the rheumatologist, the patient remained on prednisone, but the dose was reduced from 20 mg to 10 mg due to SRC. To reduce the risk of disturbances in uremic bone and mineral metabolism, alfacalcidol and calcium carbonate were introduced.

Empirical ceftriaxone was introduced to treat the infection, but it was discontinued after four days and replaced with vancomycin and levofloxacin to treat the inflammation around the exit of the CVC. Bacterial cultures were taken from inside the catheter but showed negative results. The patient suffered from insomnia and depression and was given hydroxyzine and daily sertraline. Several other prophylactic medications were started on the first day of admission, including enoxaparin, pentoxifylline, pantoprazole, allopurinol, *Lactobacillus rhamnosus*, and nystatin.

Within the second and third week of hospitalization, the patient experienced severe diarrhea and gastrointestinal pain with fever. Labs showed progressive thrombocytopenia with schistocytes present in the blood smear, anemia, and prolonged INR, without bleeding (Figure 5). Multiple transfusions of concentrates of irradiated leukocyte-depleted red blood cells were administered. Furthermore, cyclophosphamide and sertraline were terminated, and darbepoetin alfa injections were given. Moreover, an ACE inhibitor was suspended due to developing hypotension. The patient received a combination drug of tramadol + paracetamol in combination with metamizole to alleviate fever and pain. Daily meropenem was added to the antibiotic regimen. The CVC was replaced, a second culture was acquired from the tip of the catheter, and the results were again negative. Despite broad-spectrum antibiotic treatment, the inflammatory markers continued to increase, and CRP reached 56mg/l and procalcitonin 3.5µg/l (Figure 4). This led to another antibiotic adjustment with the initiation of gentamicin and linezolid. The modification of antibiotics improved the patient's condition. A positive stool culture for fungi determined the diagnosis of fungal infection of the gastrointestinal tract. Nystatin, metronidazole, and fluconazole were administered. The patient was also given methylprednisolone with an initial dose of 125 mg IV daily that was later increased to 500 mg. The patient remained severely cachectic with hypovolemia, hypoalbuminemia, marked loss of body mass, and low-calorie intake. Therefore, he received 20% albumin daily, as needed 0.9% NaCl, and one intravenous feeding with macronutrients. BP increased again, and enalapril 10 mg per day was successfully started.

**Figure 5:**
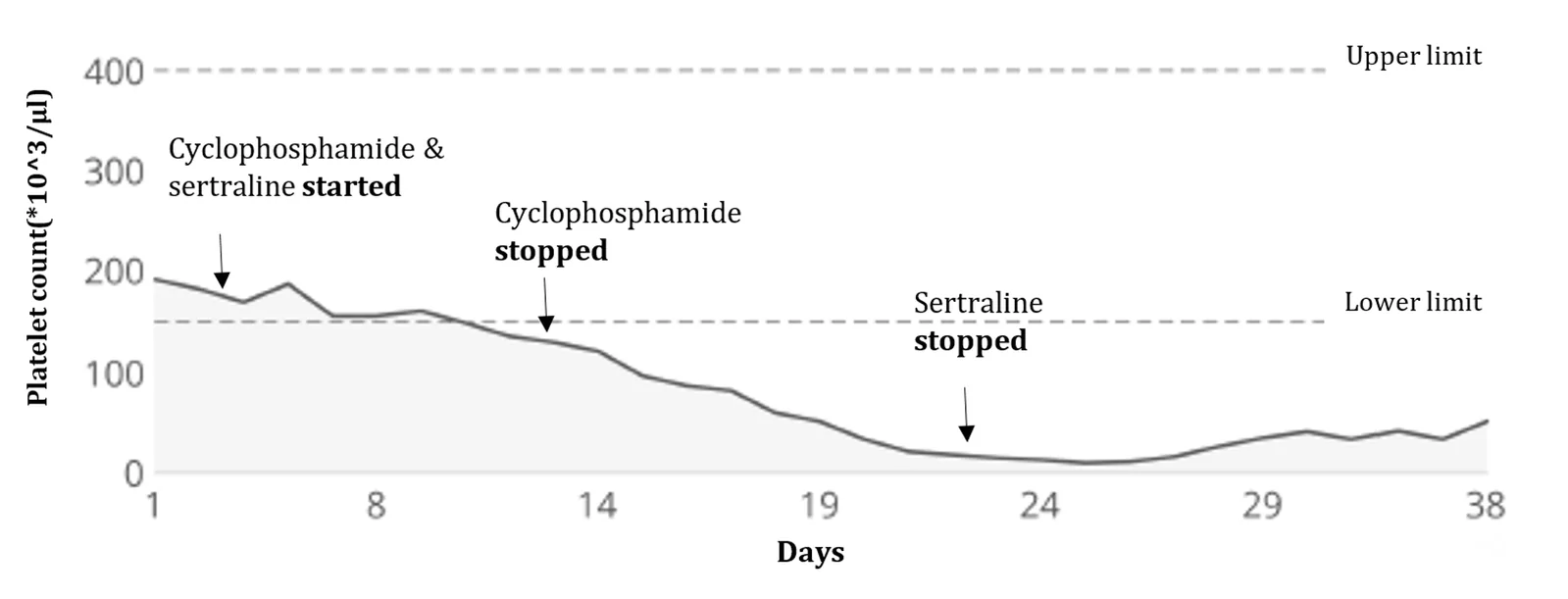
Thrombocytopenia (platelet count with reference range) during cyclophosphamide and sertraline therapy

In the consecutive week, persistent symptoms of insomnia were noticed, and zopiclone was added to the medication list. In the following days, the patient reported weakness with increasing, severe dyspnea. Oxygen therapy, 2-4 L/min, was given until the end of hospitalization. The patient developed mild pneumonia in the last week of hospitalization. To rule out COVID-19 infection, SARS-CoV-2 tests were conducted several times, and the results were always negative. Numerous *C. albicans*, *A. baumanii*, and *E. coli* were grown in the sputum culture; fluconazole and colistin were administered.

Non-pharmacological management 

The patient underwent frequent hemodialysis due to acute kidney injury (AKI) with persistent anuria and uremia, and five TPE sessions were planned for the treatment of SSc. Kaplan's equation was used to calculate the TPE dose: (0.065 x weight (kg)) x (1 - Hct) [17]. Prior to TPE, an antibody-screening test was performed to assess markers commonly present in patients with connective tissue diseases. After three sessions, TPE was stopped due to a fever of 40 ^o^C and high inflammatory markers during the second week of hospitalization. The TPE break was further prolonged as the patient was quarantined after coming into contact with a SARS-CoV-2-positive patient on the ward. A reverse transcription PCR test for COVID-19 was negative. A second course with five sessions of TPE was implemented after the inflammatory parameters decreased. Therefore, the patient received a total of eight TPE sessions (Table 2).

**Table 2:** Calculated TPE dose delivered to the patient TPE - therapeutic plasma exchange

Session	Plasma exchanged	Fresh frozen plasma
First TPE course		
I	2004 ml	2004 ml
II	2256 ml	2256 ml
III	2200 ml	2200 ml
Second TPE course		
IV	2200 ml	2200 ml
V	2300 ml	2300 ml
VI	3100 ml	2100 ml with 1000 ml 5% albumin
VII	3000 ml	2000 ml with 1000 ml 5% albumin
VIII	2980 ml	1980 ml with 1000 ml 5% albumin

Antibody tests were conducted eight days before and five days after the second course of TPE (Table 3). The initial test showed a count of scleroderma antibodies (anti-Scl-70) of 158.31 U/ml, a count of Sjogren's syndrome antibodies (anti-SS-A and anti-SS-B) of 124.79 U/ml and 138.78 U/ml, respectively, and antibodies associated with interstitial lung disease (anti-Ro52) of 39.63 U/ml. Antinuclear antibodies (ANA) levels were markedly elevated at 1:1000 when measured before TPE, and MPO-ANCA was slightly elevated, but neither was reassessed after TPE. Anti-RNA polymerase III was not measured in our patient. The second antibody test showed a reduction in the following antibody markers: anti-Scl-70 decreased to 119.63 U/ml, anti-SS-A to 109.31 U/ml, anti-SS-B to 114.14 U/ml, and anti-Ro52 to 11.73 U/ml. Hemodialysis sessions allowed removal of excess fluid (approximately 2500 ml/procedure), which led to the disappearance of lung congestion, dyspnea, edema, fluids in the pleural and peritoneal cavities, and improvement of oxygen saturation to 95%, without the need for oxygen therapy.

**Table 3:** Results of two antibody screening tests conducted before first TPE and after second TPE * The second TPE course (five sessions) TPE - therapeutic plasma exchange; Scl-70 - anti-topoisomerase I antibody; SS-A - anti-Sjogren's syndrome-related antigen A (anti-Ro); SS-B - anti-Sjogren's syndrome-related antigen B (anti-La); Ro-52 - anti-tripartite motif-containing protein 21 (TRIM21) antibody

Antibody	Before TPE	After TPE*
Scl-70	158.31	119.63
SS-A	124.79	109.31
SS-B	138.78	114.14
Ro-52	39.63	11.73

## Discussion

The prognosis of SSc is associated with the extent of organ involvement, in particular the lungs and kidneys [2]. SRC is a rare and fatal complication of SSc, and its management remains debatable. Male sex, old age of onset, and serum creatinine >265.2 µmol/L show the worst prognosis, placing our patient in a high-risk category for poor outcomes [2]. Anti-RNA polymerase III antibodies are strongly associated with an increased risk of SRC; however, this antibody was not tested in our patient, and its status therefore remains unknown [8]. SRC typically presents with acute kidney injury accompanied by new-onset or worsening hypertension with microangiopathic hemolytic anemia, thrombocytopenia, and proteinuria. The development of thrombocytopenia, anemia, and schistocytes in our patient two weeks after hospitalization raised concern for thrombotic microangiopathy (TMA). Drug-induced TMA and OS-associated TMA are both potential contributors; however, the etiology was difficult to establish given the patient's severe renal dysfunction, systemic inflammatory and infectious complications, and exposure to multiple medications.

Cardiac complications may further complicate the clinical course of SRC. Acute renal dysfunction in SRC may result in salt and water retention, contributing to pulmonary edema, particularly in patients with underlying cardiac dysfunction [18]. In addition, systemic sclerosis may cause cardiac involvement, including ventricular dysfunction, perimyocarditis, and arrhythmias [1]. In our patient, severe cardiac involvement with markedly reduced left ventricular ejection fraction further complicated the management of SRC and may have contributed to his subsequent clinical deterioration.

The purpose of SRC treatment is to reduce the extent of AKI and to restore kidney function. Therefore, hemodialysis and antihypertensive treatment play a central role in the treatment of SRC [11]. Although some studies have shown that ACE inhibitors improve or completely restore renal function and are therefore considered a key treatment for SRC, our patient required continuation of hemodialysis therapy along with chronic use of ACE inhibitor due to irreversible end-stage renal disease (ESRD) [2,11]. Immunosuppressive treatments, such as glucocorticoids (GCS), methotrexate, cyclophosphamide, and mycophenolate, are the most commonly used drugs in SSc [3,10]. However, the use of GCS for patients with SSc has its drawbacks. Studies have shown that the use of high-dose GCS (>15 mg/day) for more than six months may induce SRC [19]. Our patient has been on daily prednisone 20 mg since receiving a definitive diagnosis in 2021. We may suspect that SRC in our patient could potentially be aggravated by prolonged use of GCS. On admission to our department, the dose of prednisone was decreased to preserve residual kidney function. However, the patient developed ESRD that required chronic hemodialysis. As mentioned previously, GCS is still considered a primary approach for the management of SSc, especially in advanced stages of the disease. Therefore, the decision was made to increase the dose of prednisone. Other recommended immunosuppressive agents were not suitable for our patient, as he had mycophenolate mofetil intolerance. Furthermore, methotrexate is contraindicated for patients with renal failure, and cyclophosphamide was discontinued due to its adverse effect of myelosuppression.

Previous studies have reported potential benefits of TPE in patients with systemic sclerosis, including improvement in symptoms and organ involvement [13,15,16]. However, the clinical efficacy of TPE in SSc, particularly scleroderma renal crisis, remains uncertain. TPE may have greater potential benefit when initiated at an early stage, before irreversible organ damage develops. In our case, the patient had advanced SSc-OS involving myositis and Sjogren's syndrome, a phenotype associated with a poorer prognosis than isolated SSc [6]. Although circulating antibody titers decreased following five TPE sessions, this laboratory change was not accompanied by an observable clinical benefit. Given the complexity of the patient's presentation, the clinical significance of the reduction in antibody titers cannot be established as an effect of TPE alone.

Although ACE inhibitors remain the cornerstone of SRC management, and TPE has potential benefits, there are new emerging biomarkers and targeted therapies that may provide additional approaches to the early identification and treatment of SRC. Several urinary biomarkers, including urinary monocyte chemoattractant protein-1 (MCP-1), intercellular adhesion molecule-1 (ICAM-1), lipocalin-2, and soluble CD147, have been investigated as potential markers of renal involvement in systemic sclerosis, although their utility for early diagnosis and monitoring of SRC has not yet been established [20]. In addition, increasing evidence of complement activation in SRC has provided a rationale for complement-directed therapy, with eculizumab reported in individual cases of severe or refractory SRC. Endothelin-1 signaling represents another potential therapeutic target, and endothelin receptor antagonists, including zibotentan, have been investigated in clinical trials involving systemic sclerosis-associated kidney disease [20]. However, evidence for these emerging biomarkers and targeted therapies remains limited, and further studies are needed to determine their role in the early identification and treatment of SRC.

Despite intensive treatment, renal function did not recover, and the patient expressed a desire to be transferred to another hospital closer to his hometown. 40 days after admission to our department, he was discharged and transferred to the requested facility. Four weeks later, the patient died following worsening heart failure in the setting of thrombocytopenia, leukopenia, anemia, and myelosuppression. This case highlights the complex and potentially fatal course of SRC in a patient with advanced SSc-OS and significant multisystem involvement. The coexistence of severe renal and cardiac dysfunction, possible TMA, prolonged glucocorticoid exposure, and limited immunosuppressive treatment options complicated management and contributed to an unfavorable outcome. Early recognition of SRC, careful consideration of glucocorticoid exposure, prompt initiation of ACE inhibitor therapy, and further investigation of reliable biomarkers and targeted therapies remain important areas for improving outcomes in this high-risk population.

## Conclusions

Systemic sclerosis is a complex autoimmune disease that leads to fibrotic changes in the skin and internal organs, particularly the lungs, kidneys, and heart. Our case report highlights the challenges of managing scleroderma renal crisis in the setting of advanced systemic sclerosis overlap syndrome and severe multiorgan involvement. Although TPE resulted in a reduction in circulating antibody titers in our patient, this laboratory change did not translate into an observable clinical benefit. The patient's deterioration and death were likely multifactorial, reflecting severe heart failure, myelosuppression, and irreversible multiorgan damage. Further studies are needed to clarify the clinical efficacy, optimal timing, and patient selection for TPE, as well as emerging biomarkers and targeted therapies, in SRC associated with systemic sclerosis overlap syndrome.
